# The Costliness of Modern Treatment

**Published:** 1894-09-01

**Authors:** 


### The Hospital

An Institutional Journal of

The Medical Sciences and Hospital Administration, with

AN EXTRA NURSING SUPPLEMENT.

Vol. XVI.—No. 414.] [Sept. 1, 1894.

### The Costliness of Modern Treatment.

Is medicine a commodity for the market, or is it a scientific "light of the world," with a secular evangel and a disinterested propaganda ? It is both ; but it must be the latter rather than the former if it is in process of time to grow to that noble stature and dignity of which it is innately capable. There are, and ever will be, those in the profession who can see nothing in medicine but a marketable commodity, and who rejoice when the skilled article is scarce, and prices consequently range high. There are those, on the other hand, who never see sickness without feeling a deeply stirring impulse to relieve it, and who think that they can never be entirely happy until every human being who is sick can command for his restoration the highest resources of skilled medicine and nursing which the science of the times can afford.

The true doctor, the man with a doctor's heart, always does feel an intense desire to see the whole sick world safely "brooded under the mother wing" of an all-protecting and all-competent medicine. This is the medical instinct, the true genius of physic. But from this point of view the medical "light of the world" appeared but yesterday, and shines in its fulness at but a mere score or two of favoured places, whilst the vast world outside lies in a medical ignorance and helplessness which is all but complete.

The costliness of scientific medicine is a serious check upon its spread. As a homely illustration of this take a case which occurred within the writer's experience during the present year. A farmer's wife who had come to the climacteric was threatened with dementia and general paralysis. All kinds of medicinal treatment had been tried in the country for upwards of a year. But the patient steadily grew worse. When she was brought to London systematic massage was suggested for a considerable period. Accordingly the patient went to a private home, kept by a skilled masseuse, and paid for board, lodging, massage, and occasional medical attendance about six guineas a week. At the end of six weeks there was marked improvement, but there was also an expenditure of thirty-six guineas to be reckoned with. A family council was called, with the result that the physician in charge was asked how long he considered the treatment would have to be continued before a perfect cure could be expected. The treatment, he said, might probably require to be employed "off and on" for two years. After a few weeks' "rest" from massage, during which the patient very seriously relapsed, treatment was resumed at her own home. But even under these circumstances a nurse masseuse was constantly required, and an expenditure of three or four guineas weekly was the lowest limit of reduction. To a farmer, in these times of agricultural depression, an expenditure of three or four extra guineas a week, off and on for two years, is a matter for the consideration of many an anxious family council.

This is an illustration of the costliness of modern medicine. Many others might be offered. For example, an ovariotomist of reputation will think nothing of demanding a hundred guineas for an ovariotomy; and all other major operations are in proportion. Or a physician will order a consumptive to winter at Davos or elsewhere for three or four winters in succession. All this means money, and money in considerable sums. It means so much, indeed, that many patients to whom advice of this kind is offered will turn sorrowfully away and resolve that since nothing less costly will serve the purpose they must make up their minds to die. But the medical instinct insists that they ought not to die. Whatever be the general soundness or unsoundness of a Malthusian philosophy, medicine, when in actual conflict with an individual case of disease, or with the sickness of the whole world, has but one instinct, the instinct of cure. This is the only true medical instinct. A medicine which could contemplate plague and pestilence, disease and death, with the calm philosophy that sees an advantage to the living in the death of the sick and helpless, would be a monstrosity. It would not be a "medicine" at all. However true it may be from the statesman's or the philosopher's point of view, that a war or a pestilence may be of service to the world, from the point of view of medicine pure and simple, every sickness can only be an evil to be deplored and remedied, every premature death a calamity to be mourned, or, if possible, prevented. Medicine, as medicine, has to do with the curing of diseases and the saving of life, and with nothing else.

If this be so, the true doctor must be a propagandist. His aim is to bring to every person in the whole world who needs them, the resources of a perfected medical science and art. That is the ideal: What is the real? The reality is that not half the population, even of civilised England, can take full advantage of the highest medical resources of the times; and the chief reason for this is their costliness. But though the scientific medicine of the times is so costly, the medical profession is by no means rich. On the contrary, the vast majority of its members work steadily hard every day for pecuniary rewards which are quite inconsiderable: whilst a very numerous minority belong really to the distressed classes. It would seem then that costly as the best medical skill is, it cannot be made cheaper; and that being so, the conclusion would seem to be that the major part of the world must be content to do without it.

But that must not be. Since mankind as a whole has unitedly produced the "resources of civilisation," of which scientific medicine is one of the chief, it follows that mankind as a whole ought by right to share in those resources; and so far as medicine itself is concerned, the vast majority of doctors are most anxious to carry their science and their art into every home in every land.

One of the chief medical problems of the times is the universal diffusion of medical knowledge and tesources. The crude philanthropist tries to effect this end by means of universal medical charity. This is a two-edged weapon which injures both the recipients of the charity and the doctors at whose expense the charity is distributed. The real problem is how to bring the medical workers of the world and the medical work together on terms which shall be reciprocally honourable and advantageous. This problem, it seems to us, the general practitioner of medicine is the man to solve. He should devise means to secure for his patient, at moderate cost, the best nursing, the honestest massage, the most serviceable hydropathy, and the most competent of specialties, whilst retaining the patient under his own guidance. In other words the trained nurse, the masseur, the hydrotherapist, and the specialist should all be agents and servants of the family or general physician. This is the direction which medical development ought to take in the future. One often wonders, for instance, why a family doctor does not employ a trained nurse, who is also a masseuse and a midwife, as a dispenser or clerk. How serviceable she might be to to him in scores of emergencies, and how much money she might put into his atrophied pocket. Similarly, why should not the doctor's coachman be trained as a male masseur, or nurse, or hydrotherapeutic bathman? There is nothing derogatory to the dignity of the profession in all this. The writer remembers a country doctor of the long ago who, much to his own profit and their comfort, tried in this all-round way to supply his patients with everything therapeutical they needed, even going so far as to let out a wooden bath at a fixed charge to those old-fashioned country folk to whom a hot bath was as much a therapeutic resource, and quite as rare an experience, as the taking of a pill or a mixture. One thing is certain, that if scientific medicine is to advance, and to become universal, as it ought, it must be made sufficiently remunerative to support a cultured class of men on the one hand, and, on the other, sufficiently economical for the moderately prosperous classes to make use of it without too much pecuniary sacrifice. At present quite money enough is spent by the public in doctoring to support every legitimate practitioner in modest comfort. But unfortunately too much of it goes into the wrong pockets. It is for scientific general practitioners and physicians to put their heads together and to devise effective means for directing it into the right pockets.

